# *trans*-Dichloridobis{tris­[4-(trifluoro­methyl)phen­yl]phosphane-κ*P*}palla­dium(II) dichloro­methane monosolvate

**DOI:** 10.1107/S1600536812046971

**Published:** 2012-11-24

**Authors:** Wade L. Davis, Alfred Muller

**Affiliations:** a Research Centre for Synthesis and Catalysis, Department of Chemistry, University of Johannesburg (APK Campus), PO Box 524, Auckland Park, Johannesburg, 2006, South Africa

## Abstract

The title compound, [PdCl_2_(C_21_H_12_F_9_P)_2_]·CH_2_Cl_2_, crystallizes with two independent complex molecules (each having the Pd^II^ atom situated on an inversion centre) and a dichloro­methane molecule in the asymmetric unit. The independent Pd^II^ atoms are in perfectly linear orientations of the ligands in mutually *trans* positions, but distortions of the Cl—Pd—P angles ranging from 86.151 (19) to 93.849 (19)° are evident. The effective cone angles for the tris­[4-(trifluoro­meth­yl)phen­yl]phosphane ligand were calculated to be 159 and 161°. In the crystal, weak C—H⋯Cl/F inter­actions create a three-dimensional supramolecular network. Loose packing at two of the –CF_3_ groups resulted in large thermal vibrations which were treated as two-component disorders [occupancy ratios 0.50:0.50 and 0.628 (15):0.372 (15)].

## Related literature

For background to catalysis of palladium compounds, see: Bedford *et al.* (2004 ▶). For a description of the Cambridge Structural Database, see: Allen (2002 ▶). For background to cone angles, see: Tolman (1977 ▶); Otto (2001 ▶). For details of the conformational fit between mol­ecules using *Mercury*, see: Macrae *et al.* (2006 ▶); Weng *et al.* (2008*a* ▶,*b* ▶).
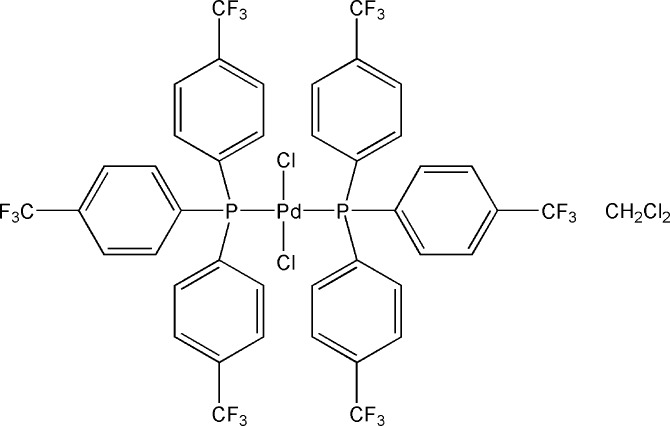


## Experimental

### 

#### Crystal data

[PdCl_2_(C_21_H_12_F_9_P)_2_]·CH_2_Cl_2_
*M* *_r_* = 1194.78
Triclinic, 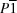

*a* = 12.1491 (10) Å
*b* = 14.0203 (13) Å
*c* = 14.4334 (13) Å
α = 72.764 (2)°
β = 78.362 (2)°
γ = 75.545 (2)°
*V* = 2252.0 (3) Å^3^
*Z* = 2
Mo *K*α radiation
μ = 0.83 mm^−1^
*T* = 100 K
0.34 × 0.31 × 0.25 mm

#### Data collection

Bruker APEX DUO 4K CCD diffractometer
Absorption correction: multi-scan (*SADABS*; Bruker, 2008 ▶) *T*_min_ = 0.766, *T*_max_ = 0.820
48517 measured reflections
11209 independent reflections
9475 reflections with *I* > 2σ(*I*)
*R*_int_ = 0.03

#### Refinement

*R*[*F* ^2^ > 2σ(*F* ^2^)] = 0.032
*wR*(*F* ^2^) = 0.077
*S* = 1.02
11209 reflections
680 parameters
116 restraints
H-atom parameters constrained
Δρ_max_ = 1.14 e Å^−3^
Δρ_min_ = −0.85 e Å^−3^

### 

Data collection: *APEX2* (Bruker, 2011 ▶); cell refinement: *SAINT* (Bruker, 2008 ▶); data reduction: *SAINT* and *XPREP* (Bruker, 2008 ▶); program(s) used to solve structure: *SIR97* (Altomare *et al.*, 1999 ▶); program(s) used to refine structure: *SHELXL97* (Sheldrick, 2008 ▶); molecular graphics: *DIAMOND* (Brandenburg & Putz, 2005 ▶); software used to prepare material for publication: *publCIF* (Westrip, 2010 ▶) and *WinGX* (Farrugia, 2012 ▶).

## Supplementary Material

Crystal structure: contains datablock(s) global, I. DOI: 10.1107/S1600536812046971/zq2189sup1.cif

Structure factors: contains datablock(s) I. DOI: 10.1107/S1600536812046971/zq2189Isup2.hkl

Additional supplementary materials:  crystallographic information; 3D view; checkCIF report

## Figures and Tables

**Table 1:** Hydrogen-bond geometry (Å, °)

*D*—H⋯*A*	*D*—H	H⋯*A*	*D*⋯*A*	*D*—H⋯*A*
C12—H12⋯Cl3	0.95	2.78	3.468 (2)	130
C7—H7*B*⋯Cl4	0.99	2.54	3.510 (4)	165
C65—H65⋯F6^i^	0.95	2.55	3.457 (3)	160
C7—H7*A*⋯Cl3^ii^	0.99	2.57	3.550 (5)	169
C15—H15⋯F17*A* ^iii^	0.95	2.52	3.341 (6)	144
C33—H33⋯F10*B*	0.95	2.53	3.435 (4)	159

Symmetry codes: (i) 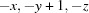; (ii) 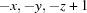; (iii) 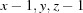.

**Fig. 1.**
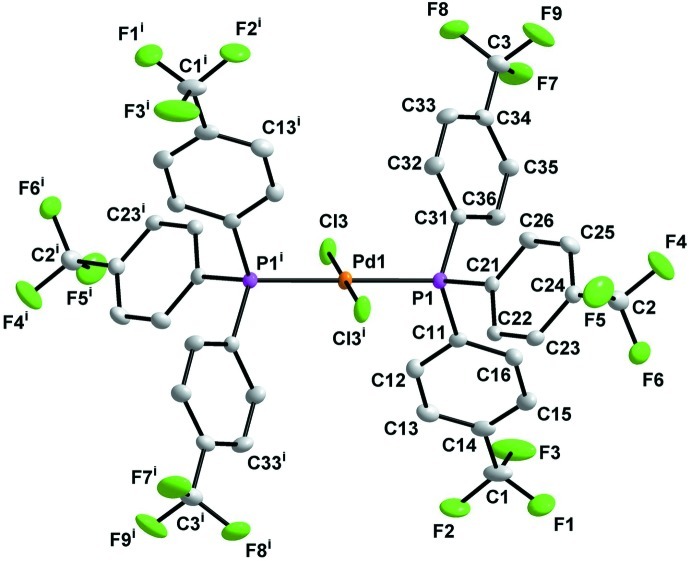
A view of molecule 1 of the title complex, showing the atom-numbering scheme and 50% probability displacement ellipsoids. Accented lettering indicate atoms generated by symmetry code (i) = -x,-y,-z. H atoms omitted for clarity.

**Fig. 2.**
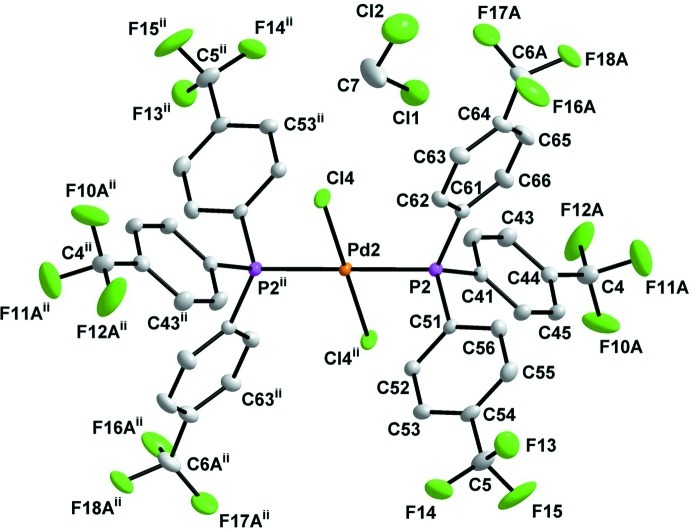
A view of molecule 2 and the dichloromethane solvate of the title complex, showing the atom-numbering scheme and 50% probability displacement ellipsoids. Accented lettering indicate atoms generated by symmetry code (ii) = 1 - x,-y,1 - z. H atoms and B component of disordered atoms omitted for clarity.

**Fig. 3.**
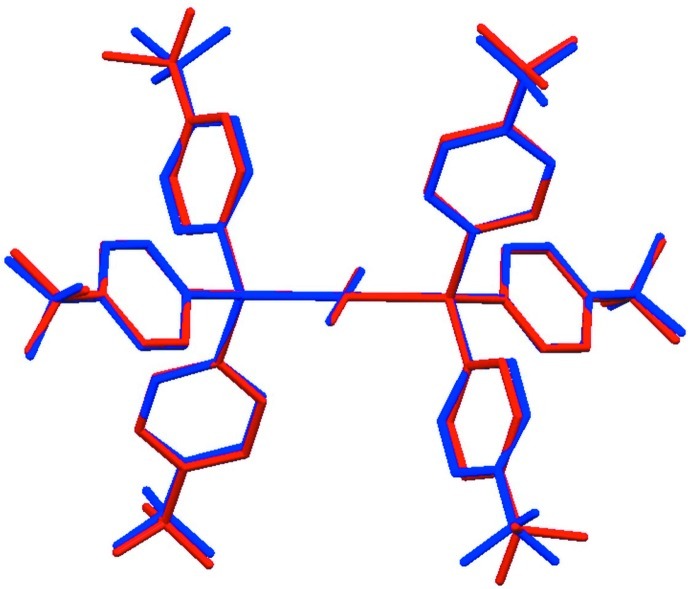
Conformational similarity between molecule 1 (blue) and molecule 2 (red) of the title complex. The root mean squared deviation; RMSD = 0.0279 Å.

## supplementary crystallographic information

### Comment

Complexes involving palladium metal centres are amongst some of the most popular catalytic precursors in organic synthesis due to their catalytic abilities. They are used in carbon-carbon bond formation reactions, *e.g.* the Heck, Stille and Suzuki reactions (Bedford *et al.*, 2004). [PdCl_2_(*L*)_2_] (*L* = tertiary phosphine, arsine or stibine) complexes can conveniently be prepared by the substitution of 1,5-cyclooctadiene (COD) from [PdCl_2_(COD)]. Reported here is the product of the reaction with tris[4-(trifluoromethyl)phenyl]phosphane ligand.

The title compound (Fig.1 and 2) crystallizes in the triclinic space group *P*1 (*Z* = 2), with the independent Pd atoms on inversion centres and each accompanied by a dichloromethane solvate molecule. Each pair of equivalent ligands is in a mutually *trans* orientation and the geometry is, therefore, perfectly linear with only slight distortions in P1—Pd1—Cl3, P2—Pd2—Cl4 angles of 87.299 (19), 92.701 (19), 93.849 (19) and 86.151 (19)°, respectively. The Pd1—P1, Pd2—P2, Pd1—Cl3 and Pd2—Cl4 bond distances of 2.3174 (5), 2.3130 (6), 2.2897 (6) and 2.2871 (6) Å, respectively fit well into the typical range for complexes of this kind (Allen, 2002). The difference between the two Pd molecules in the asymmetric unit can be illustrated by superimposing their coordination sphere coordinates (see Fig.3) using Mercury (Macrae *et al.*, 2006; Weng *et al.*, 2008*a*; Weng *et al.*, 2008*b*). This shows good fit between the two palladium molecules except in some of the CF_3_ regions.

To describe the steric demand of the phosphane ligands the Tolman cone angle (Tolman, 1977) is still the most commonly used model. Applying this model to the geometry obtained from the title compound (and adjusting the Pd—P bond distance to 2.28 Å) we calculated effective cone angles (Otto, 2001) of 161° and 159° for P1 and P2, respectively. These values are marginally larger than the average cone angle obtained from structures of this phosphane ligand in literature. Data extracted from the Cambridge Structural Database (Allen, 2002) shows an average cone angle of 154° for the phosphane from 16 hits, containing 17 useable observations with a standard deviation of ±4° and a spread from 149° to 165°. In the crystal, weak C—H···Cl/F interactions are observed with some of the CF_3_ groups showing disorder due to loose packing in these regions.

### Experimental

Tris[4-(trifluoromethyl)phenyl]phosphane (10 mg, 0.021 mmol) was dissolved in CH_2_Cl_2_ (5 cm^3^). A solution of [Pd(COD)Cl_2_] (3.10 mg, 0.011 mmol) in CH_2_Cl_2_ (5 cm^3^) was added to the phosphane solution. The mixture was stirred for 2hr at room temperature, after which the solution was left to crystallize. Orange crystals of the title compound suitable for X-ray diffraction studies were obtained in 60% yield.

^1^H NMR (CDCl_3_, 400 MHz): δ (p.p.m.) 7.67 - 7.72 (m, 12H); 7.74 - 7.81 (m, 12 H).

^31^P NMR (CDCl_3_, 162.0 MHz): δ (p.p.m.) 23.11 (s, 1P).

FTIR (cm^-1^): 2360, 1610, 1398, 1321, 1168, 1121, 1060, 1015, 955, 828, 706, 696, 633.

### Refinement

The aromatic and methylene H atoms were placed in geometrically idealized positions (C—H = 0.95 and 0.99 Å) and allowed to ride on their parent atoms, with *U*_iso_(H) = 1.2*U*_eq_(C). Two of the CF_3_ groups for molecule 2 showed large thermal vibrations and were treated to disorder refinement. The disorder models for these two CF_3_ groups were significantly different with one of the carbon atoms (C6) also modeled disordered. To keep refinement stable ellipsoid restraints (SIMU and DELU) were employed and their default refinement parameters adjusted (see _iucr_refine_instructions_details). Initially the occupancies of the two components of both CF_3_ groups were linked to free variables to refine to unity. The CF_3_ containing C4 showed an almost 50:50 distribution and in the final cycles was constrained to this. The CF_3_ containing C6 refined to a ratio of 0.628 (15):0.372 (15).

### Crystal data

**Table:**

[PdCl_2_(C_21_H_12_F_9_P)_2_]·CH_2_Cl_2_	*Z* = 2
*M**_r_* = 1194.78	*F*(000) = 1180
Triclinic, *P*1	*D*_x_ = 1.762 Mg m^−^^3^
Hall symbol: -P 1	Mo *K*α radiation, λ = 0.71073 Å
*a* = 12.1491 (10) Å	Cell parameters from 9887 reflections
*b* = 14.0203 (13) Å	θ = 2.9–28.3°
*c* = 14.4334 (13) Å	µ = 0.83 mm^−^^1^
α = 72.764 (2)°	*T* = 100 K
β = 78.362 (2)°	Cuboid, yellow
γ = 75.545 (2)°	0.34 × 0.31 × 0.25 mm
*V* = 2252.0 (3) Å^3^	

### Data collection

**Table:**

Bruker APEX DUO 4K CCD diffractometer	11209 independent reflections
Radiation source: sealed tube	9475 reflections with *I* > 2σ(*I*)
Graphite monochromator	*R*_int_ = 0.03
Detector resolution: 8.4 pixels mm^-1^	θ_max_ = 28.3°, θ_min_ = 1.5°
φ and ω scans	*h* = −16→16
Absorption correction: multi-scan (*SADABS*; Bruker, 2008)	*k* = −18→18
*T*_min_ = 0.766, *T*_max_ = 0.820	*l* = −19→19
48517 measured reflections	

### Refinement

**Table:**

Refinement on *F*^2^	Primary atom site location: structure-invariant direct methods
Least-squares matrix: full	Secondary atom site location: difference Fourier map
*R*[*F*^2^ > 2σ(*F*^2^)] = 0.032	Hydrogen site location: inferred from neighbouring sites
*wR*(*F*^2^) = 0.077	H-atom parameters constrained
*S* = 1.02	*w* = 1/[σ^2^(*F*_o_^2^) + (0.0273*P*)^2^ + 3.2668*P*] where *P* = (*F*_o_^2^ + 2*F*_c_^2^)/3
11209 reflections	(Δ/σ)_max_ = 0.001
680 parameters	Δρ_max_ = 1.14 e Å^−^^3^
116 restraints	Δρ_min_ = −0.85 e Å^−^^3^

### Special details

**Table:**

Experimental. The intensity data was collected on a Bruker Apex DUO 4 K CCD diffractometer using an exposure time of 2 s/frame. A total of 2216 frames were collected with a frame width of 0.5° covering up to θ = 28.31° with 99.8% completeness accomplished.
Geometry. All e.s.d.'s (except the e.s.d. in the dihedral angle between two l.s. planes) are estimated using the full covariance matrix. The cell e.s.d.'s are taken into account individually in the estimation of e.s.d.'s in distances, angles and torsion angles; correlations between e.s.d.'s in cell parameters are only used when they are defined by crystal symmetry. An approximate (isotropic) treatment of cell e.s.d.'s is used for estimating e.s.d.'s involving l.s. planes.
Refinement. Refinement of *F*^2^ against ALL reflections. The weighted *R*-factor *wR* and goodness of fit *S* are based on *F*^2^, conventional *R*-factors *R* are based on *F*, with *F* set to zero for negative *F*^2^. The threshold expression of *F*^2^ > σ(*F*^2^) is used only for calculating *R*-factors(gt) *etc*. and is not relevant to the choice of reflections for refinement. *R*-factors based on *F*^2^ are statistically about twice as large as those based on *F*, and *R*- factors based on ALL data will be even larger.

### Fractional atomic coordinates and isotropic or equivalent isotropic displacement parameters (Å^2^)

**Table:**

	*x*	*y*	*z*	*U*_iso_*/*U*_eq_	Occ. (<1)
C1	−0.6245 (2)	0.0991 (2)	0.0983 (2)	0.0318 (6)	
C2	−0.1366 (2)	0.4058 (2)	−0.46255 (19)	0.0303 (6)	
C3	−0.0591 (2)	0.4411 (2)	0.18078 (19)	0.0284 (5)	
C4	0.3291 (2)	0.4352 (2)	0.10003 (18)	0.0267 (4)	
C5	1.0812 (2)	0.0902 (2)	0.34731 (18)	0.0281 (5)	
C7	0.2146 (5)	0.1811 (3)	0.7105 (2)	0.0708 (13)	
H7A	0.159	0.1363	0.7429	0.085*	
H7B	0.2912	0.1363	0.7032	0.085*	
C6A	0.4634 (7)	0.4207 (7)	0.7336 (6)	0.0304 (8)	0.628 (15)
F16A	0.5626 (4)	0.4237 (5)	0.7608 (7)	0.0519 (17)	0.628 (15)
F17A	0.3961 (7)	0.3874 (3)	0.8166 (3)	0.0458 (12)	0.628 (15)
F18A	0.4156 (7)	0.5196 (12)	0.6939 (14)	0.0361 (14)	0.628 (15)
C6B	0.4798 (11)	0.4328 (12)	0.7194 (9)	0.0276 (11)	0.372 (15)
F16B	0.5757 (5)	0.4637 (8)	0.7107 (9)	0.0412 (19)	0.372 (15)
F17B	0.4512 (12)	0.3887 (6)	0.8140 (5)	0.0447 (18)	0.372 (15)
F18B	0.3952 (12)	0.514 (2)	0.700 (2)	0.0319 (18)	0.372 (15)
C11	−0.27757 (17)	0.13693 (16)	−0.00228 (15)	0.0157 (4)	
C12	−0.30036 (19)	0.04677 (17)	0.06399 (16)	0.0198 (4)	
H12	−0.2387	−0.0058	0.0881	0.024*	
C13	−0.4131 (2)	0.03313 (19)	0.09514 (17)	0.0231 (5)	
H13	−0.4282	−0.0283	0.1407	0.028*	
C14	−0.50253 (19)	0.10928 (19)	0.05941 (17)	0.0226 (5)	
C15	−0.48108 (19)	0.19894 (18)	−0.00889 (18)	0.0233 (5)	
H15	−0.5429	0.2506	−0.034	0.028*	
C16	−0.36857 (19)	0.21229 (17)	−0.03999 (17)	0.0204 (4)	
H16	−0.3535	0.2729	−0.0871	0.024*	
C21	−0.12624 (17)	0.22880 (16)	−0.16527 (15)	0.0149 (4)	
C22	−0.15437 (18)	0.18948 (16)	−0.23388 (16)	0.0173 (4)	
H22	−0.1722	0.1235	−0.2143	0.021*	
C23	−0.15639 (18)	0.24660 (17)	−0.33047 (16)	0.0187 (4)	
H23	−0.175	0.2197	−0.3772	0.022*	
C24	−0.13104 (19)	0.34358 (17)	−0.35855 (16)	0.0198 (4)	
C25	−0.1002 (2)	0.38204 (18)	−0.29173 (17)	0.0238 (5)	
H25	−0.0809	0.4475	−0.3118	0.029*	
C26	−0.0975 (2)	0.32466 (17)	−0.19515 (17)	0.0207 (4)	
H26	−0.076	0.3509	−0.1493	0.025*	
C31	−0.11086 (18)	0.24085 (16)	0.02702 (15)	0.0153 (4)	
C32	−0.00019 (19)	0.24363 (18)	0.03740 (18)	0.0228 (5)	
H32	0.0639	0.2007	0.0105	0.027*	
C33	0.0168 (2)	0.30858 (19)	0.08666 (18)	0.0251 (5)	
H33	0.0924	0.3107	0.093	0.03*	
C34	−0.0771 (2)	0.37070 (17)	0.12684 (16)	0.0195 (4)	
C35	−0.18652 (19)	0.36912 (18)	0.11652 (17)	0.0207 (4)	
H35	−0.2504	0.4125	0.1432	0.025*	
C36	−0.20374 (18)	0.30376 (17)	0.06670 (17)	0.0197 (4)	
H36	−0.2795	0.3024	0.06	0.024*	
C41	0.49547 (18)	0.23512 (16)	0.34096 (15)	0.0156 (4)	
C42	0.37650 (19)	0.25054 (18)	0.34546 (17)	0.0220 (5)	
H42	0.3328	0.2164	0.4023	0.026*	
C43	0.32186 (19)	0.31526 (19)	0.26759 (17)	0.0229 (5)	
H43	0.2409	0.3265	0.2713	0.027*	
C44	0.38674 (19)	0.36354 (17)	0.18416 (16)	0.0185 (4)	
C45	0.50467 (19)	0.34654 (17)	0.17821 (16)	0.0203 (4)	
H45	0.5484	0.3791	0.1205	0.024*	
C46	0.55962 (18)	0.28196 (17)	0.25635 (16)	0.0194 (4)	
H46	0.6408	0.2699	0.2518	0.023*	
C51	0.71490 (17)	0.13601 (16)	0.41710 (15)	0.0147 (4)	
C52	0.77923 (18)	0.04078 (17)	0.40947 (16)	0.0180 (4)	
H52	0.7416	−0.0139	0.419	0.022*	
C53	0.89861 (19)	0.02496 (18)	0.38793 (16)	0.0201 (4)	
H53	0.9421	−0.0402	0.3827	0.024*	
C54	0.95330 (18)	0.10452 (18)	0.37429 (16)	0.0200 (4)	
C55	0.8901 (2)	0.20013 (19)	0.38200 (17)	0.0234 (5)	
H55	0.9281	0.2545	0.3726	0.028*	
C56	0.77117 (19)	0.21581 (17)	0.40352 (17)	0.0205 (4)	
H56	0.728	0.2809	0.409	0.025*	
C61	0.52760 (17)	0.23591 (16)	0.53204 (15)	0.0153 (4)	
C62	0.57533 (18)	0.20004 (17)	0.61943 (16)	0.0181 (4)	
H62	0.6222	0.1337	0.6348	0.022*	
C63	0.55443 (19)	0.26092 (18)	0.68380 (17)	0.0212 (4)	
H63	0.5861	0.2362	0.7436	0.025*	
C64	0.4868 (2)	0.35857 (18)	0.66035 (18)	0.0225 (4)	
C65	0.4378 (2)	0.39403 (18)	0.57491 (19)	0.0265 (5)	
H65	0.3902	0.4601	0.5602	0.032*	
C66	0.4581 (2)	0.33302 (18)	0.51059 (18)	0.0233 (5)	
H66	0.4245	0.3575	0.4518	0.028*	
F1	−0.69349 (13)	0.13989 (14)	0.02873 (14)	0.0442 (4)	
F2	−0.63601 (13)	0.00201 (13)	0.13737 (12)	0.0388 (4)	
F3	−0.66742 (15)	0.14722 (18)	0.16893 (16)	0.0619 (6)	
F4	−0.1467 (2)	0.50458 (13)	−0.47281 (13)	0.0570 (6)	
F5	−0.04432 (16)	0.37665 (16)	−0.52398 (12)	0.0494 (5)	
F6	−0.22635 (15)	0.39581 (12)	−0.49792 (12)	0.0378 (4)	
F7	−0.15025 (15)	0.46546 (15)	0.24452 (14)	0.0475 (5)	
F8	0.02798 (14)	0.39949 (14)	0.23403 (12)	0.0388 (4)	
F9	−0.0348 (2)	0.52766 (14)	0.12020 (14)	0.0581 (6)	
F13	1.12727 (13)	0.13753 (13)	0.39375 (13)	0.0395 (4)	
F14	1.13493 (12)	−0.00744 (13)	0.36863 (13)	0.0370 (4)	
F15	1.11021 (14)	0.12869 (19)	0.25188 (12)	0.0627 (7)	
P1	−0.12923 (4)	0.15375 (4)	−0.03807 (4)	0.01399 (10)	
P2	0.55964 (4)	0.15397 (4)	0.44843 (4)	0.01338 (10)	
Cl1	0.18015 (7)	0.24502 (7)	0.59502 (6)	0.0509 (2)	
Cl2	0.21710 (9)	0.25246 (9)	0.78749 (8)	0.0671 (3)	
Cl3	−0.04796 (5)	0.00495 (4)	0.16053 (4)	0.02532 (12)	
Cl4	0.46271 (5)	0.00177 (4)	0.66133 (4)	0.02431 (12)	
Pd1	0	0	0	0.01447 (5)	
Pd2	0.5	0	0.5	0.01233 (5)	
F10A	0.3811 (5)	0.4275 (4)	0.0137 (3)	0.0651 (14)	0.5
F11A	0.3409 (4)	0.5371 (3)	0.0914 (3)	0.0521 (9)	0.5
F12A	0.2217 (3)	0.4472 (4)	0.1057 (4)	0.0647 (13)	0.5
F10B	0.2741 (4)	0.3792 (3)	0.0638 (3)	0.0485 (9)	0.5
F11B	0.2484 (5)	0.5026 (4)	0.1261 (3)	0.0696 (13)	0.5
F12B	0.3964 (4)	0.4703 (4)	0.0266 (4)	0.0546 (12)	0.5

### Atomic displacement parameters (Å^2^)

**Table:**

	*U*^11^	*U*^22^	*U*^33^	*U*^12^	*U*^13^	*U*^23^
C1	0.0218 (12)	0.0436 (15)	0.0402 (15)	−0.0142 (11)	0.0053 (10)	−0.0261 (13)
C2	0.0418 (15)	0.0267 (13)	0.0249 (13)	−0.0135 (11)	−0.0113 (11)	−0.0006 (10)
C3	0.0343 (13)	0.0273 (12)	0.0287 (13)	−0.0119 (10)	−0.0062 (11)	−0.0085 (10)
C4	0.0243 (9)	0.0319 (11)	0.0202 (9)	−0.0003 (8)	−0.0044 (8)	−0.0049 (9)
C5	0.0177 (11)	0.0429 (15)	0.0210 (12)	−0.0082 (10)	−0.0016 (9)	−0.0032 (11)
C7	0.130 (4)	0.0336 (17)	0.0309 (17)	0.007 (2)	−0.004 (2)	−0.0063 (14)
C6A	0.0347 (19)	0.0256 (18)	0.036 (2)	0.0015 (17)	−0.0091 (14)	−0.0192 (14)
F16A	0.0510 (17)	0.049 (3)	0.076 (4)	0.0070 (16)	−0.033 (2)	−0.045 (3)
F17A	0.071 (3)	0.0403 (17)	0.0263 (12)	−0.002 (2)	−0.0015 (16)	−0.0193 (11)
F18A	0.045 (3)	0.0232 (16)	0.044 (3)	0.002 (3)	−0.009 (3)	−0.0198 (12)
C6B	0.030 (2)	0.026 (3)	0.031 (2)	−0.0021 (17)	−0.005 (2)	−0.0170 (18)
F16B	0.035 (2)	0.042 (4)	0.062 (5)	−0.0056 (19)	−0.013 (2)	−0.035 (3)
F17B	0.070 (5)	0.038 (3)	0.0276 (16)	−0.004 (3)	−0.006 (3)	−0.0173 (16)
F18B	0.033 (3)	0.027 (3)	0.039 (4)	0.001 (3)	−0.003 (4)	−0.021 (2)
C11	0.0152 (9)	0.0195 (10)	0.0146 (10)	−0.0041 (8)	−0.0008 (7)	−0.0081 (8)
C12	0.0218 (10)	0.0209 (11)	0.0158 (10)	−0.0040 (9)	−0.0019 (8)	−0.0040 (8)
C13	0.0274 (12)	0.0268 (12)	0.0172 (11)	−0.0129 (10)	0.0031 (9)	−0.0068 (9)
C14	0.0190 (10)	0.0316 (12)	0.0239 (11)	−0.0094 (9)	0.0036 (9)	−0.0181 (10)
C15	0.0162 (10)	0.0251 (12)	0.0307 (13)	−0.0012 (9)	−0.0023 (9)	−0.0133 (10)
C16	0.0192 (10)	0.0179 (10)	0.0247 (11)	−0.0037 (8)	−0.0025 (9)	−0.0069 (9)
C21	0.0126 (9)	0.0153 (9)	0.0155 (10)	−0.0004 (7)	−0.0017 (7)	−0.0041 (8)
C22	0.0168 (10)	0.0157 (10)	0.0203 (10)	−0.0041 (8)	−0.0043 (8)	−0.0040 (8)
C23	0.0192 (10)	0.0210 (11)	0.0183 (10)	−0.0055 (8)	−0.0056 (8)	−0.0054 (9)
C24	0.0209 (10)	0.0190 (10)	0.0189 (11)	−0.0051 (8)	−0.0046 (8)	−0.0020 (9)
C25	0.0333 (13)	0.0188 (11)	0.0213 (11)	−0.0119 (9)	−0.0036 (10)	−0.0027 (9)
C26	0.0257 (11)	0.0207 (11)	0.0190 (11)	−0.0082 (9)	−0.0033 (9)	−0.0068 (9)
C31	0.0170 (9)	0.0153 (9)	0.0136 (9)	−0.0030 (8)	−0.0026 (8)	−0.0036 (8)
C32	0.0158 (10)	0.0266 (12)	0.0277 (12)	−0.0016 (9)	−0.0026 (9)	−0.0120 (10)
C33	0.0179 (10)	0.0302 (13)	0.0313 (13)	−0.0071 (9)	−0.0064 (9)	−0.0104 (10)
C34	0.0257 (11)	0.0188 (10)	0.0158 (10)	−0.0093 (9)	−0.0022 (8)	−0.0037 (8)
C35	0.0196 (10)	0.0227 (11)	0.0220 (11)	−0.0039 (9)	0.0004 (8)	−0.0115 (9)
C36	0.0147 (9)	0.0235 (11)	0.0239 (11)	−0.0025 (8)	−0.0022 (8)	−0.0121 (9)
C41	0.0182 (10)	0.0136 (9)	0.0152 (10)	−0.0037 (8)	−0.0027 (8)	−0.0035 (8)
C42	0.0188 (10)	0.0282 (12)	0.0180 (11)	−0.0096 (9)	−0.0006 (8)	−0.0018 (9)
C43	0.0166 (10)	0.0290 (12)	0.0232 (11)	−0.0067 (9)	−0.0038 (9)	−0.0047 (10)
C44	0.0220 (10)	0.0181 (10)	0.0162 (10)	−0.0030 (8)	−0.0038 (8)	−0.0059 (8)
C45	0.0187 (10)	0.0218 (11)	0.0159 (10)	−0.0029 (8)	0.0011 (8)	−0.0017 (9)
C46	0.0158 (10)	0.0218 (11)	0.0185 (10)	−0.0038 (8)	−0.0001 (8)	−0.0035 (9)
C51	0.0145 (9)	0.0174 (10)	0.0116 (9)	−0.0057 (8)	−0.0002 (7)	−0.0020 (8)
C52	0.0186 (10)	0.0184 (10)	0.0169 (10)	−0.0055 (8)	0.0001 (8)	−0.0045 (8)
C53	0.0184 (10)	0.0223 (11)	0.0173 (10)	−0.0014 (8)	−0.0001 (8)	−0.0054 (9)
C54	0.0148 (10)	0.0301 (12)	0.0136 (10)	−0.0064 (9)	−0.0005 (8)	−0.0028 (9)
C55	0.0217 (11)	0.0265 (12)	0.0235 (11)	−0.0123 (9)	−0.0019 (9)	−0.0035 (9)
C56	0.0206 (10)	0.0177 (10)	0.0234 (11)	−0.0055 (8)	−0.0024 (9)	−0.0048 (9)
C61	0.0151 (9)	0.0159 (10)	0.0166 (10)	−0.0058 (8)	0.0001 (8)	−0.0061 (8)
C62	0.0165 (10)	0.0171 (10)	0.0218 (11)	−0.0023 (8)	−0.0037 (8)	−0.0066 (8)
C63	0.0206 (10)	0.0247 (11)	0.0215 (11)	−0.0025 (9)	−0.0058 (9)	−0.0104 (9)
C64	0.0215 (10)	0.0222 (9)	0.0278 (10)	−0.0024 (8)	−0.0033 (8)	−0.0141 (8)
C65	0.0288 (12)	0.0183 (11)	0.0334 (13)	0.0040 (9)	−0.0100 (10)	−0.0117 (10)
C66	0.0260 (11)	0.0201 (11)	0.0240 (12)	0.0001 (9)	−0.0082 (9)	−0.0070 (9)
F1	0.0203 (7)	0.0501 (10)	0.0642 (12)	−0.0100 (7)	−0.0067 (8)	−0.0149 (9)
F2	0.0314 (8)	0.0481 (10)	0.0433 (9)	−0.0260 (7)	0.0036 (7)	−0.0121 (8)
F3	0.0365 (10)	0.0993 (17)	0.0772 (14)	−0.0365 (10)	0.0311 (9)	−0.0708 (13)
F4	0.1168 (18)	0.0246 (8)	0.0369 (10)	−0.0290 (10)	−0.0353 (11)	0.0097 (7)
F5	0.0493 (10)	0.0729 (13)	0.0185 (8)	−0.0178 (10)	−0.0010 (7)	0.0010 (8)
F6	0.0475 (9)	0.0352 (9)	0.0324 (8)	−0.0108 (7)	−0.0241 (7)	0.0032 (7)
F7	0.0410 (9)	0.0617 (12)	0.0588 (12)	−0.0089 (9)	−0.0033 (8)	−0.0473 (10)
F8	0.0395 (9)	0.0510 (10)	0.0386 (9)	−0.0146 (8)	−0.0154 (7)	−0.0194 (8)
F9	0.1088 (17)	0.0348 (10)	0.0461 (11)	−0.0419 (11)	−0.0261 (11)	−0.0006 (8)
F13	0.0216 (7)	0.0422 (9)	0.0590 (11)	−0.0112 (7)	−0.0112 (7)	−0.0119 (8)
F14	0.0181 (7)	0.0436 (9)	0.0507 (10)	0.0020 (6)	−0.0041 (7)	−0.0215 (8)
F15	0.0218 (8)	0.1211 (19)	0.0235 (8)	−0.0156 (10)	0.0034 (7)	0.0101 (10)
P1	0.0127 (2)	0.0144 (2)	0.0142 (2)	−0.00056 (19)	−0.00135 (19)	−0.0049 (2)
P2	0.0141 (2)	0.0137 (2)	0.0128 (2)	−0.00480 (19)	−0.00022 (19)	−0.00356 (19)
Cl1	0.0465 (4)	0.0494 (5)	0.0441 (4)	0.0040 (4)	−0.0071 (3)	−0.0042 (4)
Cl2	0.0607 (6)	0.0907 (8)	0.0635 (6)	−0.0249 (5)	−0.0045 (5)	−0.0351 (6)
Cl3	0.0308 (3)	0.0253 (3)	0.0136 (2)	0.0078 (2)	−0.0033 (2)	−0.0067 (2)
Cl4	0.0412 (3)	0.0232 (3)	0.0120 (2)	−0.0157 (2)	0.0024 (2)	−0.0062 (2)
Pd1	0.01427 (10)	0.01495 (11)	0.01231 (10)	0.00138 (8)	−0.00132 (8)	−0.00470 (8)
Pd2	0.01413 (10)	0.01324 (10)	0.01027 (10)	−0.00574 (8)	0.00068 (8)	−0.00314 (8)
F10A	0.082 (3)	0.067 (3)	0.0178 (14)	0.042 (2)	−0.0129 (17)	−0.013 (2)
F11A	0.077 (3)	0.0268 (13)	0.054 (2)	−0.0044 (15)	−0.040 (2)	0.0026 (14)
F12A	0.0296 (12)	0.088 (3)	0.052 (2)	−0.0220 (18)	−0.0237 (14)	0.039 (2)
F10B	0.055 (2)	0.050 (2)	0.046 (2)	−0.0195 (15)	−0.0381 (17)	0.0079 (15)
F11B	0.081 (3)	0.062 (3)	0.039 (2)	0.051 (2)	−0.0252 (18)	−0.0162 (19)
F12B	0.0292 (16)	0.076 (3)	0.037 (2)	−0.0215 (18)	−0.0146 (12)	0.033 (2)

### Geometric parameters (Å, º)

**Table:**

C1—F3	1.336 (3)	C25—H25	0.95
C1—F1	1.340 (3)	C26—H26	0.95
C1—F2	1.341 (3)	C31—C36	1.386 (3)
C1—C14	1.501 (3)	C31—C32	1.394 (3)
C2—F4	1.325 (3)	C31—P1	1.823 (2)
C2—F5	1.340 (3)	C32—C33	1.383 (3)
C2—F6	1.346 (3)	C32—H32	0.95
C2—C24	1.501 (3)	C33—C34	1.390 (3)
C3—F9	1.330 (3)	C33—H33	0.95
C3—F7	1.333 (3)	C34—C35	1.374 (3)
C3—F8	1.343 (3)	C35—C36	1.396 (3)
C3—C34	1.504 (3)	C35—H35	0.95
C4—F12B	1.255 (5)	C36—H36	0.95
C4—F12A	1.262 (4)	C41—C46	1.388 (3)
C4—F11B	1.263 (4)	C41—C42	1.398 (3)
C4—F10A	1.301 (5)	C41—P2	1.819 (2)
C4—F10B	1.408 (4)	C42—C43	1.387 (3)
C4—F11A	1.437 (4)	C42—H42	0.95
C4—C44	1.498 (3)	C43—C44	1.389 (3)
C5—F15	1.331 (3)	C43—H43	0.95
C5—F14	1.334 (3)	C44—C45	1.382 (3)
C5—F13	1.343 (3)	C45—C46	1.391 (3)
C5—C54	1.501 (3)	C45—H45	0.95
C7—Cl2	1.710 (4)	C46—H46	0.95
C7—Cl1	1.722 (4)	C51—C52	1.392 (3)
C7—H7A	0.99	C51—C56	1.398 (3)
C7—H7B	0.99	C51—P2	1.820 (2)
C6A—F17A	1.336 (7)	C52—C53	1.396 (3)
C6A—F16A	1.356 (8)	C52—H52	0.95
C6A—F18A	1.359 (15)	C53—C54	1.383 (3)
C6A—C64	1.503 (10)	C53—H53	0.95
C6B—F16B	1.312 (13)	C54—C55	1.392 (3)
C6B—F17B	1.336 (12)	C55—C56	1.390 (3)
C6B—F18B	1.34 (2)	C55—H55	0.95
C6B—C64	1.506 (17)	C56—H56	0.95
C11—C12	1.393 (3)	C61—C66	1.394 (3)
C11—C16	1.398 (3)	C61—C62	1.398 (3)
C11—P1	1.826 (2)	C61—P2	1.827 (2)
C12—C13	1.395 (3)	C62—C63	1.387 (3)
C12—H12	0.95	C62—H62	0.95
C13—C14	1.381 (3)	C63—C64	1.393 (3)
C13—H13	0.95	C63—H63	0.95
C14—C15	1.395 (3)	C64—C65	1.381 (3)
C15—C16	1.391 (3)	C65—C66	1.388 (3)
C15—H15	0.95	C65—H65	0.95
C16—H16	0.95	C66—H66	0.95
C21—C26	1.394 (3)	P1—Pd1	2.3174 (5)
C21—C22	1.398 (3)	P2—Pd2	2.3130 (6)
C21—P1	1.824 (2)	Cl3—Pd1	2.2897 (6)
C22—C23	1.388 (3)	Cl4—Pd2	2.2871 (6)
C22—H22	0.95	Pd1—Cl3^i^	2.2897 (6)
C23—C24	1.393 (3)	Pd1—P1^i^	2.3174 (5)
C23—H23	0.95	Pd2—Cl4^ii^	2.2871 (6)
C24—C25	1.384 (3)	Pd2—P2^ii^	2.3130 (6)
C25—C26	1.390 (3)		
			
F3—C1—F1	106.6 (2)	C36—C31—C32	119.3 (2)
F3—C1—F2	106.3 (2)	C36—C31—P1	121.84 (16)
F1—C1—F2	107.1 (2)	C32—C31—P1	118.88 (17)
F3—C1—C14	111.1 (2)	C33—C32—C31	120.4 (2)
F1—C1—C14	112.1 (2)	C33—C32—H32	119.8
F2—C1—C14	113.2 (2)	C31—C32—H32	119.8
F4—C2—F5	107.4 (2)	C32—C33—C34	119.8 (2)
F4—C2—F6	106.8 (2)	C32—C33—H33	120.1
F5—C2—F6	104.9 (2)	C34—C33—H33	120.1
F4—C2—C24	112.9 (2)	C35—C34—C33	120.3 (2)
F5—C2—C24	112.7 (2)	C35—C34—C3	119.6 (2)
F6—C2—C24	111.8 (2)	C33—C34—C3	120.0 (2)
F9—C3—F7	107.6 (2)	C34—C35—C36	119.9 (2)
F9—C3—F8	106.4 (2)	C34—C35—H35	120.1
F7—C3—F8	105.2 (2)	C36—C35—H35	120.1
F9—C3—C34	112.1 (2)	C31—C36—C35	120.3 (2)
F7—C3—C34	112.6 (2)	C31—C36—H36	119.8
F8—C3—C34	112.5 (2)	C35—C36—H36	119.8
F12B—C4—F12A	126.7 (3)	C46—C41—C42	119.59 (19)
F12B—C4—F11B	113.5 (4)	C46—C41—P2	122.65 (16)
F12A—C4—F11B	49.0 (3)	C42—C41—P2	117.75 (16)
F12A—C4—F10A	111.8 (4)	C43—C42—C41	120.5 (2)
F11B—C4—F10A	131.2 (4)	C43—C42—H42	119.8
F12B—C4—F10B	102.9 (4)	C41—C42—H42	119.8
F12A—C4—F10B	55.6 (3)	C42—C43—C44	119.3 (2)
F11B—C4—F10B	104.1 (4)	C42—C43—H43	120.3
F10A—C4—F10B	70.3 (4)	C44—C43—H43	120.3
F12B—C4—F11A	65.7 (3)	C45—C44—C43	120.4 (2)
F12A—C4—F11A	101.2 (4)	C45—C44—C4	119.4 (2)
F11B—C4—F11A	56.3 (4)	C43—C44—C4	120.1 (2)
F10A—C4—F11A	98.1 (4)	C44—C45—C46	120.3 (2)
F10B—C4—F11A	142.7 (3)	C44—C45—H45	119.8
F12B—C4—C44	114.6 (3)	C46—C45—H45	119.8
F12A—C4—C44	118.4 (3)	C41—C46—C45	119.8 (2)
F11B—C4—C44	112.6 (3)	C41—C46—H46	120.1
F10A—C4—C44	115.1 (3)	C45—C46—H46	120.1
F10B—C4—C44	107.9 (2)	C52—C51—C56	119.31 (19)
F11A—C4—C44	109.1 (2)	C52—C51—P2	119.73 (16)
F15—C5—F14	106.9 (2)	C56—C51—P2	120.94 (16)
F15—C5—F13	106.4 (2)	C51—C52—C53	120.5 (2)
F14—C5—F13	106.46 (19)	C51—C52—H52	119.7
F15—C5—C54	111.37 (19)	C53—C52—H52	119.7
F14—C5—C54	113.3 (2)	C54—C53—C52	119.7 (2)
F13—C5—C54	112.0 (2)	C54—C53—H53	120.2
Cl2—C7—Cl1	117.7 (2)	C52—C53—H53	120.2
Cl2—C7—H7A	107.9	C53—C54—C55	120.4 (2)
Cl1—C7—H7A	107.9	C53—C54—C5	120.5 (2)
Cl2—C7—H7B	107.9	C55—C54—C5	119.0 (2)
Cl1—C7—H7B	107.9	C56—C55—C54	119.8 (2)
H7A—C7—H7B	107.2	C56—C55—H55	120.1
F17A—C6A—F16A	105.9 (6)	C54—C55—H55	120.1
F17A—C6A—F18A	107.6 (10)	C55—C56—C51	120.2 (2)
F16A—C6A—F18A	105.3 (8)	C55—C56—H56	119.9
F17A—C6A—C64	115.6 (6)	C51—C56—H56	119.9
F16A—C6A—C64	110.6 (6)	C66—C61—C62	119.4 (2)
F18A—C6A—C64	111.2 (12)	C66—C61—P2	121.87 (17)
F16B—C6B—F17B	107.7 (11)	C62—C61—P2	118.68 (16)
F16B—C6B—F18B	108.8 (15)	C63—C62—C61	120.2 (2)
F17B—C6B—F18B	103.9 (16)	C63—C62—H62	119.9
F16B—C6B—C64	114.2 (10)	C61—C62—H62	119.9
F17B—C6B—C64	109.0 (10)	C62—C63—C64	119.6 (2)
F18B—C6B—C64	113 (2)	C62—C63—H63	120.2
C12—C11—C16	119.3 (2)	C64—C63—H63	120.2
C12—C11—P1	119.15 (16)	C65—C64—C63	120.5 (2)
C16—C11—P1	121.52 (17)	C65—C64—C6A	121.5 (4)
C11—C12—C13	120.4 (2)	C63—C64—C6A	117.9 (4)
C11—C12—H12	119.8	C65—C64—C6B	118.2 (6)
C13—C12—H12	119.8	C63—C64—C6B	120.7 (6)
C14—C13—C12	119.7 (2)	C64—C65—C66	119.9 (2)
C14—C13—H13	120.1	C64—C65—H65	120
C12—C13—H13	120.1	C66—C65—H65	120
C13—C14—C15	120.6 (2)	C65—C66—C61	120.2 (2)
C13—C14—C1	120.2 (2)	C65—C66—H66	119.9
C15—C14—C1	119.1 (2)	C61—C66—H66	119.9
C16—C15—C14	119.6 (2)	C31—P1—C21	104.08 (10)
C16—C15—H15	120.2	C31—P1—C11	106.84 (10)
C14—C15—H15	120.2	C21—P1—C11	103.49 (9)
C15—C16—C11	120.3 (2)	C31—P1—Pd1	110.60 (7)
C15—C16—H16	119.9	C21—P1—Pd1	119.18 (7)
C11—C16—H16	119.9	C11—P1—Pd1	111.70 (7)
C26—C21—C22	119.5 (2)	C41—P2—C51	108.07 (9)
C26—C21—P1	121.38 (17)	C41—P2—C61	103.66 (10)
C22—C21—P1	119.12 (16)	C51—P2—C61	103.09 (10)
C23—C22—C21	120.2 (2)	C41—P2—Pd2	110.58 (7)
C23—C22—H22	119.9	C51—P2—Pd2	111.57 (7)
C21—C22—H22	119.9	C61—P2—Pd2	119.04 (7)
C22—C23—C24	119.7 (2)	Cl3—Pd1—Cl3^i^	180
C22—C23—H23	120.2	Cl3—Pd1—P1	87.299 (19)
C24—C23—H23	120.2	Cl3^i^—Pd1—P1	92.701 (19)
C25—C24—C23	120.5 (2)	Cl3—Pd1—P1^i^	92.701 (19)
C25—C24—C2	120.3 (2)	Cl3^i^—Pd1—P1^i^	87.299 (19)
C23—C24—C2	119.2 (2)	P1—Pd1—P1^i^	180.00 (4)
C24—C25—C26	119.8 (2)	Cl4—Pd2—Cl4^ii^	180
C24—C25—H25	120.1	Cl4—Pd2—P2^ii^	86.151 (19)
C26—C25—H25	120.1	Cl4^ii^—Pd2—P2^ii^	93.849 (19)
C25—C26—C21	120.3 (2)	Cl4—Pd2—P2	93.849 (19)
C25—C26—H26	119.9	Cl4^ii^—Pd2—P2	86.151 (19)
C21—C26—H26	119.9	P2^ii^—Pd2—P2	180
			
C16—C11—C12—C13	−2.2 (3)	C5—C54—C55—C56	177.7 (2)
P1—C11—C12—C13	177.89 (17)	C54—C55—C56—C51	−0.2 (3)
C11—C12—C13—C14	0.5 (3)	C52—C51—C56—C55	0.4 (3)
C12—C13—C14—C15	1.2 (4)	P2—C51—C56—C55	178.40 (18)
C12—C13—C14—C1	−175.1 (2)	C66—C61—C62—C63	−0.5 (3)
F3—C1—C14—C13	96.0 (3)	P2—C61—C62—C63	178.88 (17)
F1—C1—C14—C13	−144.9 (2)	C61—C62—C63—C64	−0.8 (3)
F2—C1—C14—C13	−23.5 (3)	C62—C63—C64—C65	1.9 (4)
F3—C1—C14—C15	−80.4 (3)	C62—C63—C64—C6A	178.2 (4)
F1—C1—C14—C15	38.8 (3)	C62—C63—C64—C6B	−169.1 (5)
F2—C1—C14—C15	160.1 (2)	F17A—C6A—C64—C65	107.7 (7)
C13—C14—C15—C16	−1.0 (4)	F16A—C6A—C64—C65	−132.0 (6)
C1—C14—C15—C16	175.3 (2)	F18A—C6A—C64—C65	−15.3 (9)
C14—C15—C16—C11	−0.7 (3)	F17A—C6A—C64—C63	−68.5 (7)
C12—C11—C16—C15	2.4 (3)	F16A—C6A—C64—C63	51.8 (6)
P1—C11—C16—C15	−177.77 (18)	F18A—C6A—C64—C63	168.4 (6)
C26—C21—C22—C23	−1.6 (3)	F17A—C6A—C64—C6B	−176 (5)
P1—C21—C22—C23	177.88 (16)	F16A—C6A—C64—C6B	−55 (4)
C21—C22—C23—C24	−0.5 (3)	F18A—C6A—C64—C6B	61 (4)
C22—C23—C24—C25	2.1 (3)	F16B—C6B—C64—C65	−103.6 (11)
C22—C23—C24—C2	−178.4 (2)	F17B—C6B—C64—C65	135.9 (9)
F4—C2—C24—C25	−20.5 (3)	F18B—C6B—C64—C65	21.1 (14)
F5—C2—C24—C25	101.3 (3)	F16B—C6B—C64—C63	67.5 (11)
F6—C2—C24—C25	−140.9 (2)	F17B—C6B—C64—C63	−53.0 (10)
F4—C2—C24—C23	160.0 (2)	F18B—C6B—C64—C63	−167.7 (11)
F5—C2—C24—C23	−78.2 (3)	F16B—C6B—C64—C6A	147 (5)
F6—C2—C24—C23	39.6 (3)	F17B—C6B—C64—C6A	26 (3)
C23—C24—C25—C26	−1.8 (4)	F18B—C6B—C64—C6A	−89 (4)
C2—C24—C25—C26	178.7 (2)	C63—C64—C65—C66	−1.5 (4)
C24—C25—C26—C21	−0.3 (4)	C6A—C64—C65—C66	−177.7 (4)
C22—C21—C26—C25	2.0 (3)	C6B—C64—C65—C66	169.6 (5)
P1—C21—C26—C25	−177.48 (18)	C64—C65—C66—C61	0.2 (4)
C36—C31—C32—C33	−0.1 (3)	C62—C61—C66—C65	0.9 (3)
P1—C31—C32—C33	−179.78 (19)	P2—C61—C66—C65	−178.52 (19)
C31—C32—C33—C34	0.6 (4)	C36—C31—P1—C21	90.20 (19)
C32—C33—C34—C35	−1.1 (4)	C32—C31—P1—C21	−90.12 (19)
C32—C33—C34—C3	179.8 (2)	C36—C31—P1—C11	−18.9 (2)
F9—C3—C34—C35	−96.9 (3)	C32—C31—P1—C11	160.78 (17)
F7—C3—C34—C35	24.6 (3)	C36—C31—P1—Pd1	−140.65 (17)
F8—C3—C34—C35	143.3 (2)	C32—C31—P1—Pd1	39.02 (19)
F9—C3—C34—C33	82.3 (3)	C26—C21—P1—C31	8.4 (2)
F7—C3—C34—C33	−156.2 (2)	C22—C21—P1—C31	−171.03 (16)
F8—C3—C34—C33	−37.5 (3)	C26—C21—P1—C11	119.96 (18)
C33—C34—C35—C36	1.0 (3)	C22—C21—P1—C11	−59.48 (18)
C3—C34—C35—C36	−179.9 (2)	C26—C21—P1—Pd1	−115.33 (17)
C32—C31—C36—C35	0.0 (3)	C22—C21—P1—Pd1	65.23 (18)
P1—C31—C36—C35	179.66 (17)	C12—C11—P1—C31	−104.14 (18)
C34—C35—C36—C31	−0.4 (3)	C16—C11—P1—C31	76.0 (2)
C46—C41—C42—C43	−2.5 (4)	C12—C11—P1—C21	146.35 (17)
P2—C41—C42—C43	177.08 (19)	C16—C11—P1—C21	−33.5 (2)
C41—C42—C43—C44	1.0 (4)	C12—C11—P1—Pd1	16.93 (19)
C42—C43—C44—C45	0.7 (4)	C16—C11—P1—Pd1	−162.93 (16)
C42—C43—C44—C4	−178.8 (2)	C46—C41—P2—C51	−6.8 (2)
F12B—C4—C44—C45	4.0 (5)	C42—C41—P2—C51	173.62 (18)
F12A—C4—C44—C45	177.8 (4)	C46—C41—P2—C61	102.1 (2)
F11B—C4—C44—C45	−127.8 (4)	C42—C41—P2—C61	−77.44 (19)
F10A—C4—C44—C45	41.8 (5)	C46—C41—P2—Pd2	−129.21 (17)
F10B—C4—C44—C45	117.9 (3)	C42—C41—P2—Pd2	51.23 (19)
F11A—C4—C44—C45	−67.3 (3)	C52—C51—P2—C41	−110.65 (18)
F12B—C4—C44—C43	−176.5 (4)	C56—C51—P2—C41	71.37 (19)
F12A—C4—C44—C43	−2.6 (5)	C52—C51—P2—C61	140.02 (17)
F11B—C4—C44—C43	51.7 (5)	C56—C51—P2—C61	−38.0 (2)
F10A—C4—C44—C43	−138.7 (4)	C52—C51—P2—Pd2	11.13 (19)
F10B—C4—C44—C43	−62.5 (3)	C56—C51—P2—Pd2	−166.86 (15)
F11A—C4—C44—C43	112.3 (3)	C66—C61—P2—C41	5.8 (2)
C43—C44—C45—C46	−0.9 (4)	C62—C61—P2—C41	−173.60 (17)
C4—C44—C45—C46	178.6 (2)	C66—C61—P2—C51	118.38 (19)
C42—C41—C46—C45	2.2 (3)	C62—C61—P2—C51	−61.00 (18)
P2—C41—C46—C45	−177.30 (18)	C66—C61—P2—Pd2	−117.50 (18)
C44—C45—C46—C41	−0.6 (4)	C62—C61—P2—Pd2	63.12 (18)
C56—C51—C52—C53	−0.4 (3)	C31—P1—Pd1—Cl3	45.42 (7)
P2—C51—C52—C53	−178.39 (17)	C21—P1—Pd1—Cl3	165.92 (8)
C51—C52—C53—C54	0.1 (3)	C11—P1—Pd1—Cl3	−73.43 (7)
C52—C53—C54—C55	0.1 (3)	C31—P1—Pd1—Cl3^i^	−134.58 (7)
C52—C53—C54—C5	−177.7 (2)	C21—P1—Pd1—Cl3^i^	−14.08 (8)
F15—C5—C54—C53	100.5 (3)	C11—P1—Pd1—Cl3^i^	106.57 (7)
F14—C5—C54—C53	−20.0 (3)	C41—P2—Pd2—Cl4	−134.36 (8)
F13—C5—C54—C53	−140.5 (2)	C51—P2—Pd2—Cl4	105.32 (7)
F15—C5—C54—C55	−77.2 (3)	C61—P2—Pd2—Cl4	−14.56 (8)
F14—C5—C54—C55	162.2 (2)	C41—P2—Pd2—Cl4^ii^	45.64 (8)
F13—C5—C54—C55	41.8 (3)	C51—P2—Pd2—Cl4^ii^	−74.68 (7)
C53—C54—C55—C56	0.0 (3)	C61—P2—Pd2—Cl4^ii^	165.44 (8)

Symmetry codes: (i) −*x*, −*y*, −*z*; (ii) −*x*+1, −*y*, −*z*+1.

### Hydrogen-bond geometry (Å, º)

**Table:**

*D*—H···*A*	*D*—H	H···*A*	*D*···*A*	*D*—H···*A*
C12—H12···Cl3	0.95	2.78	3.468 (2)	130
C7—H7*B*···Cl4	0.99	2.54	3.510 (4)	165
C65—H65···F6^iii^	0.95	2.55	3.457 (3)	160
C7—H7*A*···Cl3^iv^	0.99	2.57	3.550 (5)	169
C15—H15···F17*A*^v^	0.95	2.52	3.341 (6)	144
C33—H33···F10*B*	0.95	2.53	3.435 (4)	159

Symmetry codes: (iii) −*x*, −*y*+1, −*z*; (iv) −*x*, −*y*, −*z*+1; (v) *x*−1, *y*, *z*−1.
